# Chloro­thia­zide–pyridine (1/3)

**DOI:** 10.1107/S1600536808014360

**Published:** 2008-05-17

**Authors:** Andrea Johnston, Alastair J. Florence, Alan R. Kennedy

**Affiliations:** a Solid-State Research Group, Strathclyde Institute of Pharmacy and Biomedical Sciences, The John Arbuthnott Building, University of Strathclyde, 27 Taylor Street, Glasgow G4 0NR, Scotland; b WestCHEM, Department of Pure & Applied Chemistry, University of Strathclyde, 295 Cathedral Street, Glasgow G1 1XL, Scotland

## Abstract

In the title compound, C_7_H_6_ClN_3_O_4_S_2_·3C_5_H_5_N, (systematic name: 6-chloro-2*H*-1,2,4-benzothia­diazine-7-sulfonamide 1,1-dioxide pyridine tris­olvate), chloro­thia­zide forms a 1:3 solvate with pyridine. The crystal structure is stabilized by strong inter­molecular N—H⋯N hydrogen bonds.

## Related literature

For details on experimental methods used to obtain this form, see: Florence *et al.* (2003 ▶, 2006 ▶). For previous studies on the non-solvated form of chloro­thaizide, see: Dupont & Dideberg (1970 ▶); Shankland *et al.* (1997 ▶). For solvated forms see: Johnston *et al.* (2007*a* ▶,*b* ▶); Johnston, Florence & Kennedy (2007 ▶); Fernandes, Florence *et al.* (2006 ▶); Fernandes, Shankland *et al.* (2007 ▶). For studies of inter­molecular inter­actions in the related thia­zide diuretic, hydro­chloro­thia­zide, see: Johnston, Florence, Shankland *et al.* (2007 ▶). For additional literature on related thia­zide compounds, see: Fabbiani *et al.* (2007 ▶); Fernandes, Johnston *et al*. (2007 ▶); Fernandes, Leech *et al.* (2007 ▶).
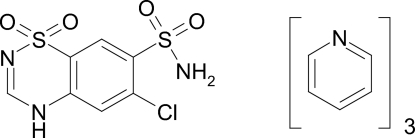


## Experimental

### 

#### Crystal data

C_7_H_6_ClN_3_O_4_S_2_·3C_5_H_5_N
*M* *_r_* = 533.02
Triclinic, 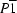

*a* = 9.0697 (15) Å
*b* = 11.863 (2) Å
*c* = 11.875 (2) Å
α = 100.691 (7)°
β = 98.667 (8)°
γ = 98.134 (7)°
*V* = 1222.1 (4) Å^3^
*Z* = 2
Mo *K*α radiation
μ = 0.37 mm^−1^
*T* = 123 (2) K
0.18 × 0.10 × 0.05 mm

#### Data collection

Nonius KappaCCD diffractometer
Absorption correction: none
14598 measured reflections
4219 independent reflections
2998 reflections with *I* > 2σ(*I*)
*R*_int_ = 0.085

#### Refinement

*R*[*F* ^2^ > 2σ(*F* ^2^)] = 0.059
*wR*(*F* ^2^) = 0.103
*S* = 1.04
4219 reflections
328 parameters
H atoms treated by a mixture of independent and constrained refinement
Δρ_max_ = 0.34 e Å^−3^
Δρ_min_ = −0.49 e Å^−3^

### 

Data collection: *DENZO* (Otwinowski & Minor, 1997 ▶) and *COLLECT* (Hooft, 1998 ▶); cell refinement: *DENZO* and *COLLECT*; data reduction: *DENZO*; program(s) used to solve structure: *SHELXS97* (Sheldrick, 2008 ▶); program(s) used to refine structure: *SHELXL97* (Sheldrick, 2008 ▶); molecular graphics: *ORTEP-3* (Farrugia, 1997 ▶); software used to prepare material for publication: *PLATON* (Spek, 2003 ▶).

## Supplementary Material

Crystal structure: contains datablocks global, I. DOI: 10.1107/S1600536808014360/bx2144sup1.cif

Structure factors: contains datablocks I. DOI: 10.1107/S1600536808014360/bx2144Isup2.hkl

Additional supplementary materials:  crystallographic information; 3D view; checkCIF report

## Figures and Tables

**Table 1:** Hydrogen-bond geometry (Å, °)

*D*—H⋯*A*	*D*—H	H⋯*A*	*D*⋯*A*	*D*—H⋯*A*
N2—H2⋯N2*S*	0.91 (4)	1.86 (4)	2.774 (4)	177 (4)
N1—H5⋯N1*S*^i^	0.85 (4)	2.07 (4)	2.900 (4)	165 (4)
N1—H6⋯N3*S*	0.83 (3)	2.13 (4)	2.946 (4)	170 (3)

Symmetry code: (i) 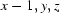.

**Fig. 1.**
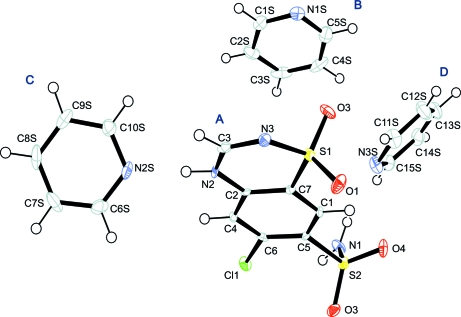
The molecular structure and atomic labelling of CT pyridine (1/3), showing 50% probablility displacement ellipsoids. 'S' in atomic labelling refers to solvent molecule.

**Fig. 2.**
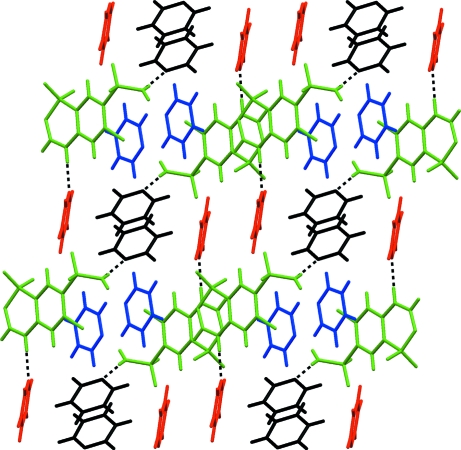
The crystal packing in CT pyridine (1/3), viewed down the a-axis. Residues A, B, C and D are coloured green, blue, red and black.

## supplementary crystallographic information

### Comment

Chlorothiazide (CT) is a thiazide diuretic drug that is known to crystallize in at least one non-solvated form (Dupont & Dideberg, 1970; Shankland *et al.*, 1997). The title compound was produced as part of an automated parallel crystallization study (Florence *et al.*, 2006) of CT as part of a wider investigation that couples automated parallel crystallization with crystal structure prediction methodology to investigate the basic science underlying the solid-state diversity of thiazide diuretics including hydrochlorothiazide (Johnston *et al.*, 2007), hydroflumethiazide (Fernandes, Johnston, A., *et al.*, 2007), trichlormethiazide (Fernandes, P., Leech, C.K. *et al.*, 2007), bendroflumethaizide (Fabbiani *et al.*, 2007) and CT. The sample was identified as a novel form using multi-sample foil transmission X-ray powder diffraction analysis (Florence *et al.*, 2003). Subsequent manual recrystallization from a saturated pyridine solution by slow evaporation at 278 K yielded a sample suitable for single-crystal X-ray diffraction (Fig. 1).

The molecules crystallize in space group *P*1 with one CT and three pyridine molecules in the asymmetric unit. The structure contains three unique N—H···N contacts (Table 1) between CT and solvent molecules whereby all hydrogen bond donors in CT, N1—H5, N1—H6 and N2—H2, are connected to a distinct pyridine molecule. The crystal structure is further stabilized by extensive offset face-to-face π^···^π interactions. All contacts combine to form a layered structure with layers comprising CT plus pyridine (residue B, Fig 1) alternating with pyridine residues C and D stacking in the [001] direction (Fig. 2).

### Experimental

A single-crystal sample of the title compound was recrystallized from a saturated pyridine solution by isothermal solvent evaporation at 278 *^o^*K.

### Refinement

The 3 H-atoms attached to N-atoms were located by difference synthesis and refined isotropically. All other H-atoms were constrained to idealized geometries using a riding model with Uĩso~(H)=1.2U~eq~(C) and C—H=0.95 \%A.

### Crystal data

**Table:**

C_7_H_6_ClN_3_O_4_S_2_·3C_5_H_5_N	*Z* = 2
*M**_r_* = 533.02	*F*_000_ = 552
Triclinic, *P*1	*D*_x_ = 1.448 Mg m^−^^3^
Hall symbol: P -1	Mo *K*α radiation λ = 0.71073 Å
*a* = 9.0697 (15) Å	Cell parameters from 3968 reflections
*b* = 11.863 (2) Å	θ = 1.0–27.1º
*c* = 11.875 (2) Å	µ = 0.37 mm^−^^1^
α = 100.691 (7)º	*T* = 123 (2) K
β = 98.667 (8)º	Cut fragment, colourless
γ = 98.134 (7)º	0.18 × 0.10 × 0.05 mm
*V* = 1222.1 (4) Å^3^	

### Data collection

**Table:**

Nonius KappaCCD diffractometer	2998 reflections with *I* > 2σ(*I*)
Radiation source: fine-focus sealed tube	*R*_int_ = 0.085
Monochromator: graphite	θ_max_ = 25.3º
*T* = 123(2) K	θ_min_ = 1.8º
φ and ω scans	*h* = −10→0
Absorption correction: none	*k* = −13→14
14598 measured reflections	*l* = −13→14
4219 independent reflections	

### Refinement

**Table:**

Refinement on *F*^2^	H atoms treated by a mixture of independent and constrained refinement
Least-squares matrix: full	*w* = 1/[σ^2^(*F*_o_^2^) + (0.0253*P*)^2^ + 1.8286*P*] where *P* = (*F*_o_^2^ + 2*F*_c_^2^)/3
*R*[*F*^2^ > 2σ(*F*^2^)] = 0.059	(Δ/σ)_max_ < 0.001
*wR*(*F*^2^) = 0.103	Δρ_max_ = 0.34 e Å^−^^3^
*S* = 1.04	Δρ_min_ = −0.49 e Å^−^^3^
4219 reflections	Extinction correction: none
328 parameters	

### Special details

**Table:**

Geometry. All e.s.d.'s (except the e.s.d. in the dihedral angle between two l.s. planes) are estimated using the full covariance matrix. The cell e.s.d.'s are taken into account individually in the estimation of e.s.d.'s in distances, angles and torsion angles; correlations between e.s.d.'s in cell parameters are only used when they are defined by crystal symmetry. An approximate (isotropic) treatment of cell e.s.d.'s is used for estimating e.s.d.'s involving l.s. planes.
Refinement. Refinement of *F*^2^ against ALL reflections. The weighted *R*-factor *wR* and goodness of fit *S* are based on *F*^2^, conventional *R*-factors *R* are based on *F*, with *F* set to zero for negative *F*^2^. The threshold expression of *F*^2^ > σ(*F*^2^) is used only for calculating *R*-factors(gt) *etc*. and is not relevant to the choice of reflections for refinement. *R*-factors based on *F*^2^ are statistically about twice as large as those based on *F*, and *R*- factors based on ALL data will be even larger.

### Fractional atomic coordinates and isotropic or equivalent isotropic displacement parameters (Å^2^)

**Table:**

	*x*	*y*	*z*	*U*_iso_*/*U*_eq_	
Cl1	−0.11042 (9)	0.34675 (7)	0.54702 (7)	0.0161 (2)	
S1	0.24543 (9)	−0.02698 (7)	0.33227 (7)	0.0127 (2)	
S2	−0.16390 (10)	0.25714 (7)	0.26176 (7)	0.0134 (2)	
O3	0.3649 (3)	0.0349 (2)	0.2890 (2)	0.0213 (6)	
O1	0.1454 (3)	−0.1213 (2)	0.2501 (2)	0.0230 (6)	
O2	−0.1320 (3)	0.20934 (19)	0.14895 (19)	0.0185 (6)	
O4	−0.3120 (2)	0.2257 (2)	0.28715 (19)	0.0197 (6)	
N3	0.3205 (3)	−0.0768 (2)	0.4406 (2)	0.0145 (7)	
N2	0.2459 (3)	0.0587 (2)	0.5857 (3)	0.0121 (7)	
N1	−0.1235 (4)	0.3939 (3)	0.2847 (3)	0.0178 (7)	
C3	0.3177 (4)	−0.0285 (3)	0.5481 (3)	0.0137 (8)	
H3	0.3730	−0.0587	0.6068	0.016*	
C2	0.1548 (3)	0.1084 (3)	0.5117 (3)	0.0093 (7)	
C7	0.1389 (3)	0.0736 (3)	0.3908 (3)	0.0098 (7)	
C1	0.0429 (3)	0.1218 (3)	0.3176 (3)	0.0101 (7)	
H1	0.0320	0.0968	0.2356	0.012*	
C5	−0.0367 (3)	0.2054 (3)	0.3622 (3)	0.0098 (7)	
C6	−0.0171 (4)	0.2406 (3)	0.4846 (3)	0.0108 (8)	
C4	0.0752 (4)	0.1928 (3)	0.5575 (3)	0.0103 (7)	
H4	0.0851	0.2173	0.6396	0.012*	
N1S	0.7057 (3)	0.5432 (2)	0.4150 (3)	0.0195 (7)	
C1S	0.6985 (4)	0.5848 (3)	0.5263 (3)	0.0202 (9)	
H1S	0.7624	0.5608	0.5850	0.024*	
C2S	0.6037 (4)	0.6605 (3)	0.5610 (3)	0.0242 (9)	
H2S	0.6020	0.6870	0.6413	0.029*	
C3S	0.5120 (4)	0.6967 (3)	0.4769 (4)	0.0291 (10)	
H3S	0.4469	0.7500	0.4981	0.035*	
C4S	0.5158 (4)	0.6547 (3)	0.3615 (4)	0.0308 (10)	
H4S	0.4520	0.6769	0.3014	0.037*	
C5S	0.6150 (4)	0.5793 (3)	0.3352 (3)	0.0255 (9)	
H5S	0.6186	0.5516	0.2555	0.031*	
N2S	0.2984 (3)	0.1270 (2)	0.8267 (2)	0.0200 (7)	
C6S	0.2000 (4)	0.1232 (3)	0.9002 (3)	0.0237 (9)	
H6S	0.0953	0.1160	0.8698	0.028*	
C7S	0.2439 (5)	0.1292 (3)	1.0180 (3)	0.0335 (11)	
H7S	0.1705	0.1246	1.0669	0.040*	
C8S	0.3955 (5)	0.1419 (3)	1.0634 (3)	0.0319 (11)	
H8S	0.4288	0.1467	1.1441	0.038*	
C9S	0.4979 (4)	0.1475 (3)	0.9891 (3)	0.0263 (10)	
H9S	0.6035	0.1570	1.0177	0.032*	
C10S	0.4444 (4)	0.1391 (3)	0.8729 (3)	0.0237 (9)	
H10S	0.5159	0.1420	0.8223	0.028*	
N3S	0.1270 (3)	0.4933 (3)	0.1813 (3)	0.0245 (8)	
C11S	0.2125 (4)	0.4257 (3)	0.1287 (3)	0.0302 (10)	
H11S	0.2221	0.3542	0.1517	0.036*	
C12S	0.2882 (4)	0.4540 (4)	0.0421 (3)	0.0336 (10)	
H12S	0.3486	0.4034	0.0071	0.040*	
C13S	0.2739 (4)	0.5576 (4)	0.0079 (3)	0.0313 (10)	
H13S	0.3233	0.5792	−0.0520	0.038*	
C14S	0.1872 (4)	0.6288 (3)	0.0617 (3)	0.0261 (9)	
H14S	0.1760	0.7008	0.0403	0.031*	
C15S	0.1165 (4)	0.5939 (3)	0.1478 (3)	0.0222 (9)	
H15S	0.0573	0.6440	0.1851	0.027*	
H2	0.261 (4)	0.083 (3)	0.665 (3)	0.034 (12)*	
H5	−0.166 (4)	0.433 (3)	0.334 (3)	0.036 (13)*	
H6	−0.046 (4)	0.420 (3)	0.262 (3)	0.014 (10)*	

### Atomic displacement parameters (Å^2^)

**Table:**

	*U*^11^	*U*^22^	*U*^33^	*U*^12^	*U*^13^	*U*^23^
Cl1	0.0195 (5)	0.0159 (5)	0.0153 (5)	0.0021 (4)	0.0067 (4)	0.0090 (4)
S1	0.0133 (5)	0.0150 (5)	0.0108 (5)	0.0021 (4)	0.0024 (4)	0.0065 (4)
S2	0.0141 (5)	0.0152 (5)	0.0120 (5)	0.0059 (4)	−0.0002 (4)	0.0045 (4)
O3	0.0187 (14)	0.0303 (15)	0.0237 (15)	0.0147 (12)	0.0141 (12)	0.0108 (11)
O1	0.0245 (15)	0.0209 (14)	0.0180 (14)	−0.0067 (11)	−0.0053 (11)	0.0089 (11)
O2	0.0274 (14)	0.0209 (14)	0.0083 (13)	0.0029 (10)	0.0014 (11)	0.0101 (11)
O4	0.0137 (13)	0.0261 (14)	0.0191 (14)	0.0106 (11)	−0.0027 (11)	0.0014 (11)
N3	0.0125 (16)	0.0176 (16)	0.0158 (18)	0.0070 (13)	0.0019 (13)	0.0067 (13)
N2	0.0136 (16)	0.0164 (16)	0.0066 (18)	0.0049 (13)	−0.0016 (13)	0.0037 (13)
N1	0.0214 (19)	0.0163 (17)	0.022 (2)	0.0100 (14)	0.0121 (16)	0.0064 (15)
C3	0.0083 (18)	0.0125 (18)	0.020 (2)	0.0083 (16)	0.0000 (16)	−0.0016 (15)
C2	0.0046 (17)	0.0105 (17)	0.012 (2)	0.0037 (14)	0.0000 (15)	−0.0015 (14)
C7	0.0072 (18)	0.0103 (17)	0.011 (2)	0.0013 (14)	0.0008 (15)	−0.0012 (14)
C1	0.0107 (18)	0.0127 (17)	0.0054 (19)	0.0002 (14)	0.0006 (15)	−0.0001 (14)
C5	0.0070 (18)	0.0113 (18)	0.010 (2)	0.0038 (14)	−0.0009 (15)	−0.0006 (14)
C6	0.0084 (18)	0.0083 (17)	0.016 (2)	0.0009 (14)	0.0062 (15)	0.0008 (14)
C4	0.0108 (18)	0.0115 (17)	0.0081 (19)	0.0037 (14)	0.0016 (15)	−0.0013 (14)
N1S	0.0182 (17)	0.0141 (16)	0.026 (2)	0.0021 (14)	0.0053 (15)	0.0029 (13)
C1S	0.020 (2)	0.016 (2)	0.024 (2)	0.0078 (17)	−0.0011 (17)	−0.0004 (16)
C2S	0.022 (2)	0.018 (2)	0.032 (2)	0.0007 (17)	0.0116 (19)	−0.0018 (17)
C3S	0.015 (2)	0.012 (2)	0.060 (3)	0.004 (2)	0.009 (2)	0.0043 (17)
C4S	0.019 (2)	0.026 (2)	0.044 (3)	0.017 (2)	−0.011 (2)	−0.0025 (18)
C5S	0.028 (2)	0.023 (2)	0.023 (2)	0.0068 (18)	0.0018 (19)	−0.0024 (18)
N2S	0.0232 (19)	0.0243 (18)	0.0105 (17)	0.0013 (13)	−0.0039 (15)	0.0077 (14)
C6S	0.018 (2)	0.021 (2)	0.026 (3)	−0.0027 (17)	0.0002 (18)	−0.0032 (17)
C7S	0.042 (3)	0.034 (2)	0.021 (3)	0.0019 (18)	0.017 (2)	−0.011 (2)
C8S	0.053 (3)	0.025 (2)	0.012 (2)	0.0080 (17)	−0.002 (2)	−0.006 (2)
C9S	0.027 (2)	0.022 (2)	0.025 (3)	0.0052 (17)	−0.011 (2)	0.0027 (18)
C10S	0.024 (2)	0.029 (2)	0.019 (2)	0.0038 (17)	0.0054 (19)	0.0084 (18)
N3S	0.031 (2)	0.0234 (18)	0.0208 (19)	0.0043 (14)	0.0106 (15)	0.0060 (15)
C11S	0.038 (3)	0.024 (2)	0.031 (3)	0.0078 (18)	0.008 (2)	0.0084 (19)
C12S	0.036 (3)	0.041 (3)	0.029 (3)	0.007 (2)	0.016 (2)	0.012 (2)
C13S	0.033 (2)	0.043 (3)	0.019 (2)	0.009 (2)	0.011 (2)	−0.001 (2)
C14S	0.033 (2)	0.024 (2)	0.021 (2)	0.0096 (17)	0.0016 (19)	0.0002 (19)
C15S	0.026 (2)	0.020 (2)	0.019 (2)	0.0021 (17)	0.0019 (18)	0.0035 (17)

### Geometric parameters (Å, °)

**Table:**

Cl1—C6	1.730 (3)	C2S—H2S	0.9500
S1—O3	1.435 (2)	C3S—C4S	1.376 (6)
S1—O1	1.439 (2)	C3S—H3S	0.9500
S1—N3	1.613 (3)	C4S—C5S	1.384 (5)
S1—C7	1.749 (3)	C4S—H4S	0.9500
S2—O4	1.434 (2)	C5S—H5S	0.9500
S2—O2	1.443 (2)	N2S—C10S	1.332 (4)
S2—N1	1.575 (3)	N2S—C6S	1.340 (4)
S2—C5	1.785 (3)	C6S—C7S	1.381 (5)
N3—C3	1.304 (4)	C6S—H6S	0.9500
N2—C3	1.342 (4)	C7S—C8S	1.376 (5)
N2—C2	1.383 (4)	C7S—H7S	0.9500
N2—H2	0.91 (4)	C8S—C9S	1.377 (5)
N1—H5	0.85 (4)	C8S—H8S	0.9500
N1—H6	0.83 (3)	C9S—C10S	1.372 (5)
C3—H3	0.9500	C9S—H9S	0.9500
C2—C4	1.393 (4)	C10S—H10S	0.9500
C2—C7	1.398 (4)	N3S—C11S	1.330 (5)
C7—C1	1.392 (4)	N3S—C15S	1.338 (4)
C1—C5	1.381 (4)	C11S—C12S	1.384 (5)
C1—H1	0.9500	C11S—H11S	0.9500
C5—C6	1.413 (4)	C12S—C13S	1.381 (5)
C6—C4	1.368 (4)	C12S—H12S	0.9500
C4—H4	0.9500	C13S—C14S	1.371 (5)
N1S—C5S	1.331 (5)	C13S—H13S	0.9500
N1S—C1S	1.337 (4)	C14S—C15S	1.381 (5)
C1S—C2S	1.380 (5)	C14S—H14S	0.9500
C1S—H1S	0.9500	C15S—H15S	0.9500
C2S—C3S	1.372 (5)		
			
?···?	?		
			
O3—S1—O1	116.53 (15)	C3S—C2S—C1S	118.5 (4)
O3—S1—N3	108.29 (14)	C3S—C2S—H2S	120.8
O1—S1—N3	108.71 (14)	C1S—C2S—H2S	120.8
O3—S1—C7	108.12 (14)	C2S—C3S—C4S	119.0 (4)
O1—S1—C7	109.09 (14)	C2S—C3S—H3S	120.5
N3—S1—C7	105.55 (15)	C4S—C3S—H3S	120.5
O4—S2—O2	119.51 (14)	C3S—C4S—C5S	118.3 (4)
O4—S2—N1	108.62 (17)	C3S—C4S—H4S	120.8
O2—S2—N1	108.54 (16)	C5S—C4S—H4S	120.8
O4—S2—C5	106.13 (14)	N1S—C5S—C4S	124.0 (4)
O2—S2—C5	104.48 (14)	N1S—C5S—H5S	118.0
N1—S2—C5	109.16 (16)	C4S—C5S—H5S	118.0
C3—N3—S1	121.8 (2)	C10S—N2S—C6S	116.7 (3)
C3—N2—C2	123.4 (3)	N2S—C6S—C7S	123.0 (4)
C3—N2—H2	115 (2)	N2S—C6S—H6S	118.5
C2—N2—H2	122 (2)	C7S—C6S—H6S	118.5
S2—N1—H5	118 (3)	C8S—C7S—C6S	119.0 (4)
S2—N1—H6	115 (2)	C8S—C7S—H7S	120.5
H5—N1—H6	125 (3)	C6S—C7S—H7S	120.5
N3—C3—N2	127.6 (3)	C7S—C8S—C9S	118.5 (4)
N3—C3—H3	116.2	C7S—C8S—H8S	120.7
N2—C3—H3	116.2	C9S—C8S—H8S	120.7
N2—C2—C4	120.0 (3)	C10S—C9S—C8S	118.6 (4)
N2—C2—C7	120.9 (3)	C10S—C9S—H9S	120.7
C4—C2—C7	119.1 (3)	C8S—C9S—H9S	120.7
C1—C7—C2	120.1 (3)	N2S—C10S—C9S	124.1 (3)
C1—C7—S1	120.3 (2)	N2S—C10S—H10S	118.0
C2—C7—S1	119.6 (2)	C9S—C10S—H10S	118.0
C5—C1—C7	121.2 (3)	C11S—N3S—C15S	116.9 (3)
C5—C1—H1	119.4	N3S—C11S—C12S	123.5 (4)
C7—C1—H1	119.4	N3S—C11S—H11S	118.2
C1—C5—C6	117.7 (3)	C12S—C11S—H11S	118.2
C1—C5—S2	118.0 (2)	C13S—C12S—C11S	118.4 (4)
C6—C5—S2	124.2 (2)	C13S—C12S—H12S	120.8
C4—C6—C5	121.7 (3)	C11S—C12S—H12S	120.8
C4—C6—Cl1	117.8 (2)	C14S—C13S—C12S	118.9 (3)
C5—C6—Cl1	120.5 (2)	C14S—C13S—H13S	120.6
C6—C4—C2	120.1 (3)	C12S—C13S—H13S	120.6
C6—C4—H4	119.9	C13S—C14S—C15S	118.7 (3)
C2—C4—H4	119.9	C13S—C14S—H14S	120.6
C5S—N1S—C1S	116.3 (3)	C15S—C14S—H14S	120.6
N1S—C1S—C2S	124.0 (3)	N3S—C15S—C14S	123.4 (3)
N1S—C1S—H1S	118.0	N3S—C15S—H15S	118.3
C2S—C1S—H1S	118.0	C14S—C15S—H15S	118.3
			
O3—S1—N3—C3	104.1 (3)	C1—C5—C6—C4	−1.1 (4)
O1—S1—N3—C3	−128.4 (3)	S2—C5—C6—C4	176.0 (2)
C7—S1—N3—C3	−11.5 (3)	C1—C5—C6—Cl1	178.9 (2)
S1—N3—C3—N2	5.9 (5)	S2—C5—C6—Cl1	−4.1 (4)
C2—N2—C3—N3	3.1 (5)	C5—C6—C4—C2	0.9 (5)
C3—N2—C2—C4	175.3 (3)	Cl1—C6—C4—C2	−179.0 (2)
C3—N2—C2—C7	−3.5 (5)	N2—C2—C4—C6	−178.8 (3)
N2—C2—C7—C1	177.9 (3)	C7—C2—C4—C6	0.0 (4)
C4—C2—C7—C1	−0.9 (4)	C5S—N1S—C1S—C2S	0.4 (5)
N2—C2—C7—S1	−4.4 (4)	N1S—C1S—C2S—C3S	−0.7 (5)
C4—C2—C7—S1	176.8 (2)	C1S—C2S—C3S—C4S	1.2 (5)
O3—S1—C7—C1	72.7 (3)	C2S—C3S—C4S—C5S	−1.4 (5)
O1—S1—C7—C1	−54.9 (3)	C1S—N1S—C5S—C4S	−0.6 (5)
N3—S1—C7—C1	−171.6 (2)	C3S—C4S—C5S—N1S	1.1 (5)
O3—S1—C7—C2	−105.0 (3)	C10S—N2S—C6S—C7S	−1.1 (5)
O1—S1—C7—C2	127.4 (3)	N2S—C6S—C7S—C8S	1.3 (6)
N3—S1—C7—C2	10.7 (3)	C6S—C7S—C8S—C9S	−0.4 (6)
C2—C7—C1—C5	0.8 (4)	C7S—C8S—C9S—C10S	−0.6 (5)
S1—C7—C1—C5	−176.9 (2)	C6S—N2S—C10S—C9S	0.0 (5)
C7—C1—C5—C6	0.2 (4)	C8S—C9S—C10S—N2S	0.8 (6)
C7—C1—C5—S2	−177.0 (2)	C15S—N3S—C11S—C12S	−0.5 (6)
O4—S2—C5—C1	117.4 (2)	N3S—C11S—C12S—C13S	−0.4 (6)
O2—S2—C5—C1	−9.8 (3)	C11S—C12S—C13S—C14S	0.8 (6)
N1—S2—C5—C1	−125.7 (3)	C12S—C13S—C14S—C15S	−0.4 (6)
O4—S2—C5—C6	−59.7 (3)	C11S—N3S—C15S—C14S	0.9 (5)
O2—S2—C5—C6	173.2 (3)	C13S—C14S—C15S—N3S	−0.5 (6)
N1—S2—C5—C6	57.2 (3)		

### Hydrogen-bond geometry (Å, °)

**Table:**

*D*—H···*A*	*D*—H	H···*A*	*D*···*A*	*D*—H···*A*
N2—H2···N2S	0.91 (4)	1.86 (4)	2.774 (4)	177 (4)
N1—H5···N1S^i^	0.85 (4)	2.07 (4)	2.900 (4)	165 (4)
N1—H6···N3S	0.83 (3)	2.13 (4)	2.946 (4)	170 (3)

Symmetry codes: (i) *x*−1, *y*, *z*.
